# Supplementary material for: Serum Advanced Glycation End Products and Their Soluble Receptor as New Biomarkers in Systemic Lupus Erythematosus
Source: Biomedicines. 2024 Mar 7;12(3):610. doi: 10.3390/biomedicines12030610 (PMC10968350; doi:10.3390/biomedicines12030610)
Supplement: Supplementary file 1 [file biomedicines-12-00610-s001.zip › biomedicines-2887779-supplementary.pdf]

SUPPLEMENTARY MATERIAL

Study	SLE:HC	SLE vs HC	Type of AGE	AGEs vs SLE characteristics
**Serum AGEs**				
**Rodríguez-García1998** [59]	37:57	**No**	Pentosidine	–
**Nienhuis2008** [20]	10:10	**No**	CEL, CML	No differences
**Nowak2021** [23]	31:26	**Only as a group**	CEL, CML, pentosidine	–
**Ene2021** [24]	38 LN<br>44 SLE non-LN<br>40 HC	**Higher in SLE**	Pentosidine	–
**Nisihara2021** [21]	79 SLE<br>No HC	–	Pentosidine	**Lower pentosidine in skin discoid lesions and photosensitivity**<br>No differences in SLEDAI or SDI
**Plasma AGEs**				
**Chen2015** [22]	36:16	**Higher in SLE**	AGEs	**Positive correlation with SLEDAI**

Supplementary Table S1: Previous works in the literature studying advanced glycation end-products (AGEs) in systemic lupus erythematosus (SLE) and their observations. *HC: healthy controls; IMT: intimate media thickness; CEL: Nξ-(carboxyethyl)lysine; CML: Nξ-(carboxymethyl)lysine; SLEDAI: SLE disease activity index; LN: lupus nephritis; SDI: SLE damage index*

Study	SLE:HC	SLE vs HC	sRAGE vs SLE characteristics
**Serum sRAGE**			
**Nienhuis2008** [20]	10:10	**Higher in quiescent SLE vs HC**	**Higher in active disease**
**Lee2013** [29]	60 APS (35 SLE)	**Higher in APS**	–
**Bayoumy2013** [26]	82 proliferative LN<br>53 non-proliferative LN<br>43 mixed LN<br>No HC	–	**Lower in patients with poor response to therapy**
**Bobek2014** [25]	19:28	**Lower in SLE**	**Positive correlation with C4**
**Manganelli2019** [30]	60:22	**Higher in SLE**	No difference in activity markers
**Ene2021** [24]	38 LN<br>44 SLE non-LN<br>40 HC	**Lower in SLE**	–
**Plasma sRAGE**			
**Ma2012** [27]	120:40	**Lower in SLE**	**Higher in patients with skin rash, serositis or longer treatment**<br>**Negative correlation with WBC, lymphocytes, and neutrophils**
**Yu2015** [28]	105:43	**Lower in SLE**	**Higher in patients with longer treatment**
**Okuyucu2022** [60]	27:24	**Lower in SLE**	**Negative correlation with SLEDAI and lower patients with flare**

Supplementary Table S2: Previous works in the literature studying the soluble receptor for advanced glycation end-products (sRAGE) in systemic lupus erythematosus (SLE) and their observations. *HC: healthy controls; APS: antiphospholipid syndrome; LN: lupus nephritis; WBC: white blood count; SLEDAI: SLE disease activity index.*

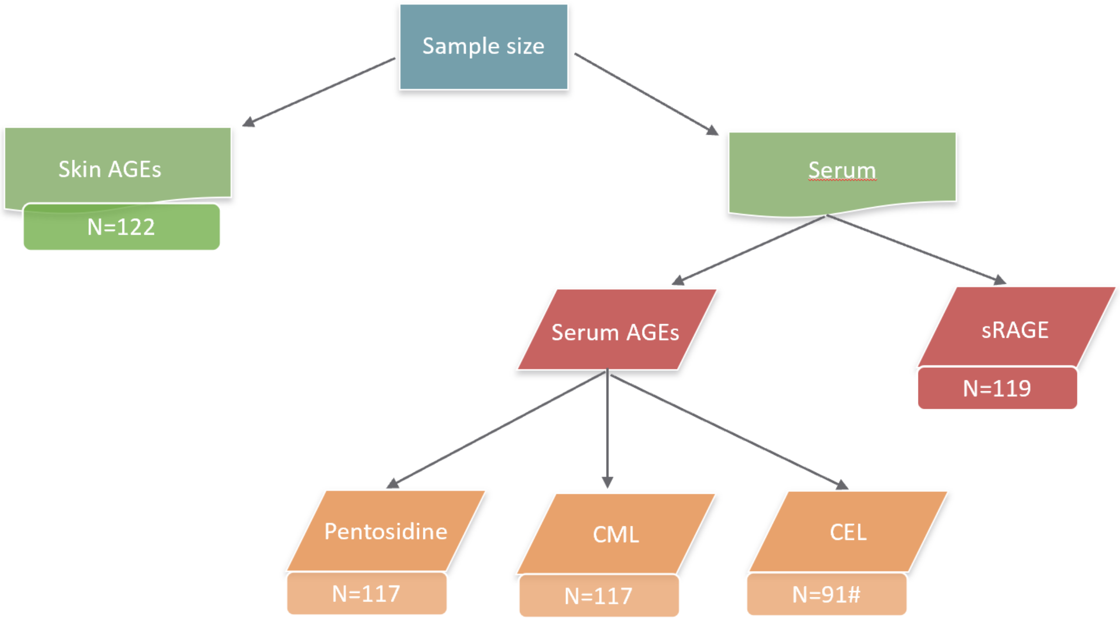

Supplementary Figure S1: Sample size for each of the analysis. *For the comparison between patients with SLE and HC only 62 SLE patients could be included due to a younger mean age than HC and impossibility to age-match a higher number. Also, SLE patients with cardiovascular risk factors had to be excluded due to it being a criterion of exclusion in the HC. #: The sample size for analyzing CEL is reduced due to a shortage of the same ELISA kit. *AGEs: advanced glycation end-products; SLE: systemic lupus erythematosus; HC: healthy controls; sRAGE: soluble receptor for advanced glycation end-products; CML: Nξ-(carboxymethyl)lysine; CEL: Nξ-(car-boxyethyl)lysine.*

Demographics like age, gender, and ethnicity.

Disease characteristics: All the criteria gathered in the 1997 ACR and 2012 SLICC SLE classificatory criteria were recorded, as well as other characteristics such as different ab or overlap with other SADs.

- Disease duration was assessed as a continuous variable and divided into 4 groups: 0-5, 6-10, 11-20 and ≥ 20 years since SLE diagnosis.
- Leukocyturia and hematuria were divided into 5 categories according to number of leukocytes/red blood cells detected per field (S0: none, S1: 0-5, S2: 5-10, S3: 10-20, S4: 20-50, S5: >50/camp).
- Number of manifestations ever: the accumulated number of manifestations according to those included in the ACR or SLICC SLE classificatory criteria.

Treatment: all SLE treatments that patients were receiving at the moment of inclusion were recorded. We created 3 groups with treatment regimens progressively more intense: receiving no treatment or only glucocorticoids vs on antimalarials ± glucocorticoids vs on immunosuppressants/biological drugs ± antimalarials ± glucocorticoids. We also recorded treatments for CVRF like antihypertensive or dyslipidemia drugs, antiplatelet or anticoagulant therapy.

Different indexes for measuring SLE activity and accrual damage:

- Physician global assessment (PGA): divided into <1, [1-3], >3 according to the sample's distribution.
- Patient global assessment (PtGA) ≤ 3 vs >3, categorized according to the nonlinear association observed in the scatter plot.
- Disease Activity Score 28 (DAS28): remission ≤ 2.6, low (2.6-3.2], moderate (3.2-5.1] and high activity >5.1.
- SLE disease activity index 2000 (SLEDAI 2-K): remission=0, mild [0-4], moderate (4-11], severe >11. For statistical purposes we grouped patients in remission and with mild activity.
- SLE disease activity score (SLE-DAS) as a continuous variable. Remission ≤2.08; mild activity, (2.08-7.64]; moderate/severe activity>7.64.
- SLICC/American College of Rheumatology (SLICC/ACR) Damage Index (SDI), analyzed both as a quantitative variable, categorized in two groups (SDI 0 vs ≥ 1), and in three groups (0 vs 1 vs ≥ 1).
- IL-6 was measured using a Sandwich ELISA Kit provided by R&D Systems (Human IL-6 Quantikine, D6050); sensitivity 0.70 pg/mL; precision measured as coefficient of variation < 8% (intra-assay), < 10% (inter-assay).

PROs like the Health Assessment Questionnaire (HAQ) (divided into normal<0.3, mild [0.3-1.3], moderate (1.3-1.8], severe >1.8), the Functional Assessment of Chronic Illness Therapy – Fatigue Scale (FACIT), and patient global assessment by a visual analogic scale (PtGA).

Cardiovascular variables:

- CVRF: presence of at least one: obesity (BMI > 30 Kg/m²), AHT, DLP, CRD or hyperuricaemia. The smoking status was considered as a separate variable due to its high correlation with AGEs levels.
- Cardiovascular events (CVE): angina, myocardial infarction, cerebrovascular accident, peripheral arterial disease, intestinal ischemia, or ischemia of some other territory). CVRF&CVE indicates the presence of either/both CVRF or CVE (CVRF&CVE>0) or the sum (CVRF&CVE).
- CVE_SDI: cardiovascular events assessed in the SDI (cerebral vascular accident, pulmonary infarction, angina or coronary bypass, myocardial infarction, venous thrombosis, or infarction of the gastrointestinal tract).

Supplementary Figure S2: Variables collected and their classification. *ACR: American College of Rheumatology; SLE: systemic lupus erythematosus, SLICC: systemic lupus International Collaborating Clinics, CVRF: cardiovascular risk factors; ab: antibodies; SADs: systemic autoimmune diseases; BMI: body mass index; AHT: arterial hypertensio; DLP: dislipidemia; CRD: chronic renal disease; AGEs: advance glycation end-products.*

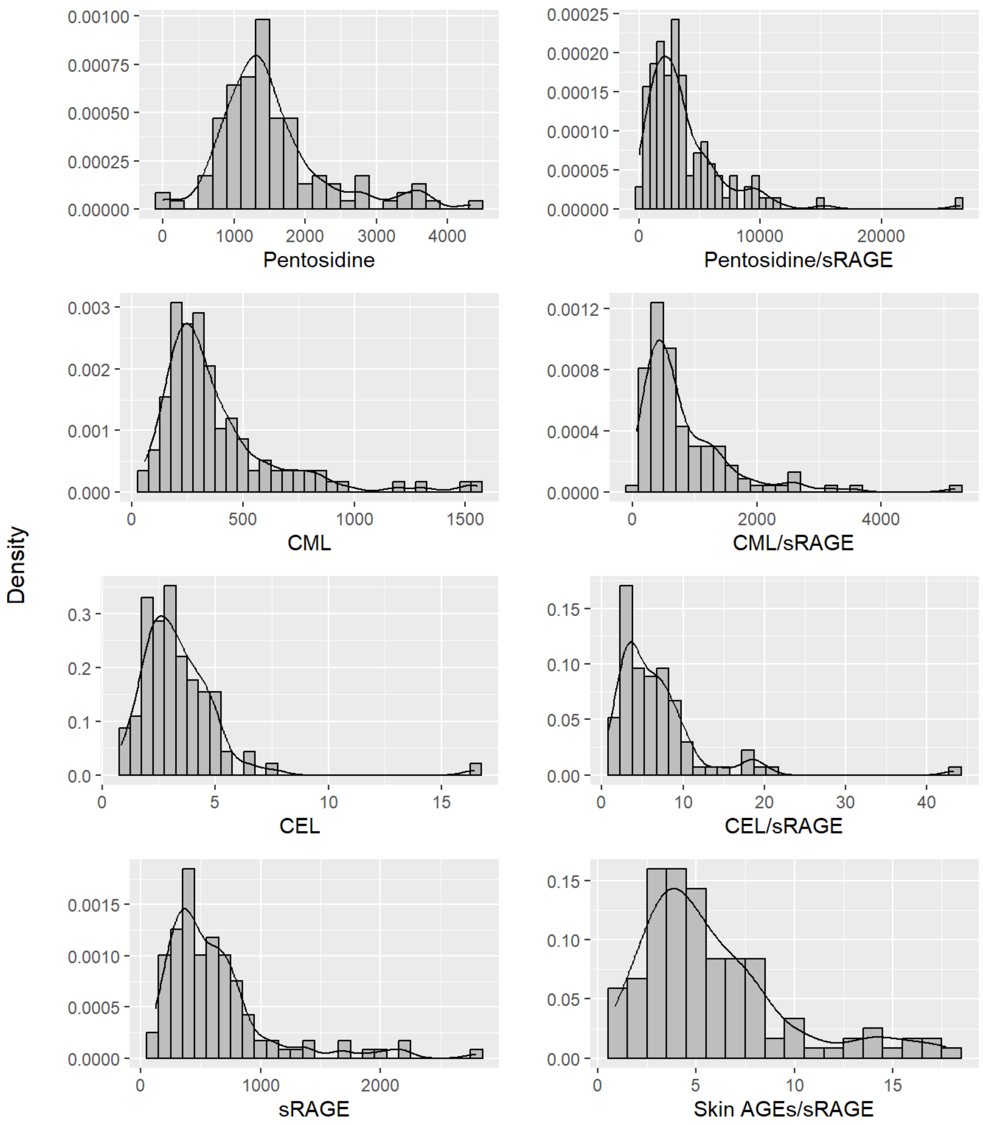

Supplementary Figure S1: Density graphics of serum advanced glycation end-products, their soluble receptor and their ratios showing their right-skewed distribution. *CML: Nξ-(carboxymethyl)lysine; CEL: Nξ-(carboxyethyl)lysine; sRAGE: soluble receptor for advanced glycation end-products.*

Variables	First tertile [0.1180)	Second tertile [1180,1594)	Third tertile [1594,4334]	p-value
	N=39	*N=39*	*N=39*	
Gender: Female	36 (92.3%)	36 (92.3%)	37 (94.9%)	0.815
Age	52.1 (14.8)	49.5 (15.5)	51.1 (14.9)	0.453
Body mass index	25.7 (5.03)	25.9 (4.54)	24.9 (4.87)	0.474
Ethnicity				0.992
Caucasian	29 (74.4%)	25 (64.1%)	25 (64.1%)	
Latin	8 (20.5%)	8 (20.5%)	11 (28.2%)	
Other	2 (5.13%)	6 (15.4%)	3 (7.69%)	
Years of duration	8.00 [4.00;13.5]	10.0 [2.50;17.5]	10.0 [1.00;18.5]	0.994
Smoker	11 (28.2%)	9 (23.1%)	10 (25.6%)	0.723
Classificatory Criteria and Other Clinical and Serological Data				
Constitutional symptoms	4 (10.3%)	4 (10.3%)	3 (7.69%)	0.207
Cutaneous	25 (64.1%)	34 (87.2%)	28 (71.8%)	0.725
Photosensitivity	21 (53.8%)	25 (64.1%)	25 (64.1%)	0.305
Oral ulcer	17 (43.6%)	17 (43.6%)	14 (35.9%)	0.160
Alopecia	13 (33.3%)	20 (51.3%)	19 (48.7%)	0.883
Arthritis	28 (71.8%)	32 (82.1%)	29 (74.4%)	0.346
Serositis	5 (12.8%)	4 (10.3%)	1 (2.56%)	0.593
Renal	3 (7.69%)	4 (10.3%)	1 (2.56%)	0.228
Neurological	4 (10.3%)	3 (7.69%)	3 (7.69%)	0.258
Hematological	33 (84.6%)	26 (66.7%)	24 (61.5%)	0.333
ANA+ ever	39 (100%)	39 (100%)	39 (100%)	
Anti-dsDNA+ ever	23 (59.0%)	26 (66.7%)	25 (64.1%)	0.179
Anti-Sm+ ever	8 (20.5%)	10 (25.6%)	3 (7.69%)	0.199
Anti-Ro60+ ever	16 (41.0%)	19 (48.7%)	13 (33.3%)	0.899
Anti-Ro52+ ever	11 (28.2%)	9 (23.1%)	7 (17.9%)	0.186
Low complement	20 (51.3%)	22 (56.4%)	20 (51.3%)	0.210
Cardiac	1 (2.56%)	1 (2.56%)	2 (5.13%)	0.110
Raynaud	14 (35.9%)	12 (31.6%)	13 (33.3%)	0.417
APL antibodies carrier	8 (20.5%)	10 (25.6%)	9 (23.1%)	0.305
APS	1 (2.56%)	3 (7.69%)	1 (2.56%)	0.733
Other SADs	24 (61.5%)	23 (59%)	28 (71.8%)	0.100
Serological Variables				
CRP*	0.14 [0.08;0.32]	0.18 [0.08;0.42]	0.13 [0.08;0.31]	0.869
ESR*	13.0 [7.00;21.0]	10.0 [5.00;18.0]	9.00 [2.00;18.5]	0.448
Anti-dsDNA+*	4.00 [2.00;15.8]	5.00 [1.00;12.0]	3.00 [1.00;12.5]	0.551
CH50*	58.7 [42.0;67.5]	60.3 [48.2;72.8]	62.2 [56.4;70.9]	0.109
C3*	105 (22.6)	105 (22.4)	109 (23.5)	0.671
C4*	18.8 (8.18)	20.4 (9.80)	20.5 (6.99)	0.349
IL-6*	2.90 [1.93;4.66]	1.98 [1.55;3.53]	2.32 [1.40;3.63]	0.598
SLE Activity and Damage Indexes				
DAS28	2.36 [1.68;3.11]	2.02 [1.37;2.76]	2.10 [1.33;3.25]	0.698
SLEDAI	4.00 [2.00;6.00]	4.00 [2.00;6.00]	4.00 [2.00;7.00]	0.255
SLE-DAS	4.18 [1.78;7.28]	1.79 [1.20;6.15]	2.53 [0.82;4.86]	0.087
SDI	0.00 [0.00;1.00]	1.00 [0.00;1.00]	0.00 [0.00;1.00]	0.587
PGA	2.00 [1.00;3.00]	2.00 [1.00;2.50]	2.00 [1.00;3.00]	0.565
Patient Reported Outcomes				
FACIT	18.0 [13.0;28.0]	18.0 [10.0;27.0]	16.0 [9.50;26.0]	0.386
HAQ	0.50 [0.00;0.94]	0.38 [0.00;0.88]	0.31 [0.00;0.72]	0.795
PtGA	2.50 [1.00;5.00]	3.00 [1.00;4.50]	3.00 [1.00;4.75]	0.368
Pain VAS	2.00 [0.00;6.25]	2.00 [0.00;5.00]	3.00 [0.00;6.00]	0.926
Comorbidities and Cardiovascular Disease				
Hypertension	9 (23.1%)	9 (23.1%)	8 (20.5%)	0.929
Dyslipidemia	6 (15.4%)	3 (7.69%)	3 (7.69%)	0.850
Cardiovascular disease	1 (2.56%)	3 (7.69%)	1 (2.56%)	0.706

Variables	First tertile [0.1180)	Second tertile [1180,1594)	Third tertile [1594,4334]	p-value
Chronic renal disease	1 (2.56%)	1 (2.56%)	1 (2.56%)	0.129
Hyperuricemia	0 (0.00%)	2 (5.13%)	0 (0.00%)	0.887
Obesity	9 (23.1%)	8 (20.5%)	5 (12.8%)	0.360
CVRF >0	15 (38.5%)	17 (43.6%)	15 (38.5%)	0.816
CVRF				0.579
0	24 (61.5%)	22 (56.4%)	24 (61.5%)	
1	8 (20.5%)	12 (30.8%)	13 (33.3%)	
2	4 (10.3%)	4 (10.3%)	2 (5.13%)	
3	3 (7.69%)	1 (2.56%)	0 (0.00%)	
CVE				0.249
0	36 (92.3%)	33 (84.6%)	37 (94.9%)	
1	3 (7.69%)	4 (10.3%)	0 (0.00%)	
2	0 (0.00%)	1 (2.56%)	1 (2.56%)	
3	0 (0.00%)	1 (2.56%)	1 (2.56%)	
CVE_SDI_presence	2 (5.13%)	5 (12.8%)	2 (5.13%)	0.944
CVRF&CVE >0	15 (38.5%)	17 (43.6%)	16 (41.0%)	0.518
CVRF & CVE				0.795
0	24 (61.5%)	22 (56.4%)	23 (59.0%)	
1	12 (30.8%)	11 (28.2%)	14 (35.9%)	
2	3 (7.69%)	4 (10.3%)	1 (2.56%)	
3	0 (0.00%)	1 (2.56%)	0 (0.00%)	
4	0 (0.00%)	1 (2.56%)	1 (2.56%)	
Treatments				
Dyslipidemia drugs	5 (12.8%)	5 (12.8%)	4 (10.3%)	0.749
Antihypertensives	9 (23.1%)	10 (25.6%)	9 (23.1%)	0.976
Antimalarials	28 (71.8%)	32 (82.1%)	29 (74.4%)	0.493
cDMARD	6 (15.4%)	6 (15.4%)	4 (10.3%)	0.591
bDMARD	2 (5.13%)	3 (7.69%)	1 (2.56%)	0.432
Mycophenolic acid	9 (23.1%)	6 (15.4%)	4 (10.3%)	0.493
Cyclosporine: 0	39 (100%)	39 (100%)	39 (100%)	
Azathioprine	8 (20.5%)	3 (7.69%)	7 (17.9%)	0.933
Cyclophosphamide: 0	39 (100%)	39 (100%)	39 (100%)	
Treatment				0.494
Others	3 (7.69%)	5 (12.8%)	6 (15.4%)	
Antimalarials	14 (35.9%)	19 (48.7%)	18 (46.2%)	
Immunosuppressants	22 (56.4%)	15 (38.5%)	15 (38.5%)	
AGEs				
Skin AGEs	2.61 (0.61)	2.55 (0.69)	2.43 (0.66)	0.214
CML	277 [199;365]	320 [194;497]	294 [234;460]	0.372
CEL	3.14 [2.33;4.18]	3.16 [2.04;3.73]	2.82 [2.38;4.33]	0.485
sRAGE	505 [369;790]	447 [317;751]	556 [341;703]	0.342

Supplementary Table S3: Non-significant (p-value >0.1) demographic and disease characteristics of systemic lupus erythematosus patients and their distribution according to pentosidine tertiles in the exploratory analysis. "c" indicates variables which have been categorized as previously stated in the methodology section. * Indicates values according to the blood test performed in the study. *ANA: antinuclear antibodies; APL: antiphospholipid; APS; antiphospholipid syndrome; SADs: systemic autoimmune diseases; CRP: C-reactive protein; ESR: Erythrocyte sedimentation rate; CH50, C3 and C4: Complement CH50, C3 and C4; IL-6: interleukin 6; DAS28: disease activity score 28; SLEDAI: systemic lupus erythematosus disease activity index; SLE-DAS: systemic lupus erythematosus disease activity score; SDI: systemic lupus erythematosus damage index; PGA: physician global assessment; FACIT: functional assessment of chronic illness therapy; HAQ: health assessment questionnaire disability index; PtGA: patient global assessment; VAS: visual analogic scale; CVRF: cardiovascular risk factors (obesity = IMC> 30 Kg/m2, arterial hypertension, dyslipidemia, chronic renal disease or hyperuricaemia); CVE:*

cardiovascular events (angina, myocardial infarction, cerebrovascular accident, peripheral arterial disease, intestinal ischemia or ischemia of some other territory); CVE_SDI: cardiovascular events assessed in the SLE damage index (cerebral vascular accident, pulmonary infarction, angina or coronary bypass, myocardial infarction, venous thrombosis or infarction of the gastrointestinal tract); cDMARD; Disease-modifying antirheumatic drugs; bDMARD: biological DMARD; AGEs: advanced glycation end products; CML: Nξ-(carboxymethyl)lysine; CEL: Nξ-(carboxyethyl)lysine; sRAGE: receptor for advanced glycation end-products.

	Estimate	2.5%	97.5%	t-value	p-value
(Intercept)	1481.3214	1341.8941	1620.7487	21.0447	0.0000
Pulmonary	1181.8786	507.4192	1856.3379	3.4710	**0.0007**

Supplementary Table S4: Linear regression model showing associations between pentosidine levels and systemic lupus erythematosus variables. We only show the results that were statistically significant indicated by $p<0.05$ (bold).

**Variables**	**First tertile [57.6, 240)**	**Second tertile [239.8, 383)**	**Third tertile [382.9,1555]**	**p-value**
	N=39	*N=39*	*N=39*	
Gender: Female	34 (87.2%)	37 (94.9%)	38 (97.4%)	0.417
Age	50.3 (16.2)	52.0 (14.3)	50.3 (14.7)	0.496
Body mass index	25.5 (5.23)	25.9 (5.01)	24.8 (4.20)	0.311
Smoker	11 (28.2%)	12 (30.8%)	8 (20.5%)	0.127
Classificatory Criteria and Other Clinical and Serological Data				
Constitutional symptoms	3 (7.69%)	3 (7.69%)	4 (10.3%)	0.564
Cutaneous	32 (82.1%)	24 (61.5%)	32 (82.1%)	0.566
Photosensitivity	22 (56.4%)	25 (64.1%)	27 (69.2%)	0.883
Oral ulcers	20 (51.3%)	12 (30.8%)	17 (43.6%)	0.281
Alopecia	17 (43.6%)	13 (33.3%)	24 (61.5%)	0.926
Arthritis	27 (69.2%)	29 (74.4%)	33 (84.6%)	0.459
Serositis	3 (7.69%)	4 (10.3%)	2 (5.13%)	0.554
Neurological	3 (7.69%)	4 (10.3%)	3 (7.69%)	0.899
Hematological	24 (61.5%)	31 (79.5%)	28 (71.8%)	0.480
ANA+ ever	39 (100%)	39 (100%)	39 (100%)	
Anti-dsDNA+ ever	19 (48.7%)	28 (71.8%)	26 (66.7%)	0.587
Anti-Sm+ ever	7 (17.9%)	4 (10.3%)	10 (25.6%)	0.205
Anti-Ro60+ ever	16 (41.0%)	13 (33.3%)	19 (48.7%)	0.106
Anti-Ro52+ ever	10 (25.6%)	7 (17.9%)	10 (25.6%)	0.208
Low complement	21 (53.8%)	21 (53.8%)	19 (48.7%)	0.939
Direct Coombs +	1 (4.55%)	4 (16.7%)	4 (19.0%)	0.242
Pulmonary	0 (0.00%)	2 (5.13%)	2 (5.13%)	0.587
Cardiac	0 (0.00%)	3 (7.69%)	1 (2.56%)	0.981
Raynaud	13 (33.3%)	14 (35.9%)	12 (30.8%)	0.286
APL antibodies carrier	10 (25.6%)	11 (28.2%)	8 (20.5%)	0.594
APS	2 (5.13%)	1 (2.56%)	2 (5.13%)	0.887
Other SADs	24 (61.5%)	26 (66.7%)	23 (59.0%)	0.713
Serological Variables				
CRP*	0.14 [0.08;0.38]	0.16 [0.07;0.30]	0.12 [0.08;0.34]	0.497
ESR*	9.00 [4.25;20.0]	9.00 [5.00;16.8]	13.0 [7.50;20.5]	0.120
Anti-dsDNA+*	2.00 [1.00;9.00]	3.00 [1.00;11.8]	6.00 [2.00;20.5]	0.675
Anti-dsDNA>RV*	7 (17.9%)	11 (28.9%)	18 (46.2%)	0.135
CH50*	67.2 [53.0;72.2]	60.6 [53.1;68.2]	58.6 [43.2;66.3]	0.366
C3*	107 (22.2)	108 (21.3)	104 (24.7)	0.216
C4*	19.5 (8.43)	20.3 (7.18)	19.8 (9.47)	0.848
IL-6*	2.28 [1.29;3.23]	2.08 [1.84;3.01]	3.43 [1.86;4.69]	0.176
SLE Activity and Damage Indexes				
DAS28	2.11 [1.28;3.09]	2.10 [1.47;2.85]	2.43 [1.74;3.31]	0.270
SLEDAI	4.00 [2.00;6.00]	4.00 [2.00;6.00]	5.00 [2.00;7.00]	0.393
SLE-DAS	2.55 [1.20;6.19]	3.55 [1.01;5.59]	2.53 [1.78;7.18]	0.470
SDI	0.00 [0.00;1.00]	0.00 [0.00;1.00]	0.00 [0.00;1.00]	0.297
Patient Reported Outcomes				
FACIT	15.0 [9.50;26.5]	20.0 [12.5;30.5]	18.0 [10.0;25.5]	0.930
HAQ	0.25 [0.00;1.00]	0.50 [0.00;0.94]	0.31 [0.03;0.59]	0.870
PtGA	2.50 [1.00;5.00]	3.00 [0.00;5.00]	3.00 [1.50;5.00]	0.974
Pain VAS	2.00 [0.00;6.25]	3.00 [0.00;6.00]	2.00 [0.00;5.50]	0.810
Comorbidities and Cardiovascular Disease				
Hypertension	7 (17.9%)	10 (25.6%)	9 (23.1%)	0.536
Dyslipidemia	4 (10.3%)	2 (5.13%)	6 (15.4%)	0.136
Cardiovascular disease	2 (5.13%)	1 (2.56%)	2 (5.13%)	0.879
Chronic renal disease	1 (2.56%)	1 (2.56%)	1 (2.56%)	0.953
Hyperuricemia	0 (0.00%)	1 (2.56%)	1 (2.56%)	0.609
Obesity	7 (17.9%)	9 (23.1%)	6 (15.4%)	0.421
CVRF >0	12 (30.8%)	18 (46.2%)	17 (43.6%)	0.568

Variables	First tertile [57.6, 240)	Second tertile [239.8, 383)	Third tertile [382.9,1555]	p-value
CVRF				0.312
0	27 (69.2%)	21 (53.8%)	22 (56.4%)	
1	6 (15.4%)	14 (35.9%)	13 (33.3%)	
2	5 (12.8%)	3 (7.69%)	2 (5.13%)	
3	1 (2.56%)	1 (2.56%)	2 (5.13%)	
CVE				0.160
0	35 (89.7%)	37 (94.9%)	34 (87.2%)	
1	4 (10.3%)	1 (2.56%)	2 (5.13%)	
2	0 (0.00%)	0 (0.00%)	2 (5.13%)	
3	0 (0.00%)	1 (2.56%)	1 (2.56%)	
CVE_SDI_presence	2 (5.13%)	2 (5.13%)	5 (12.8%)	0.848
CVRF&CVE >0	12 (30.8%)	18 (46.2%)	18 (46.2%)	0.417
CVRF&CVE				0.512
0	27 (69.2%)	21 (53.8%)	21 (53.8%)	
1	8 (20.5%)	16 (41.0%)	13 (33.3%)	
2	4 (10.3%)	1 (2.56%)	3 (7.69%)	
3	0 (0.00%)	0 (0.00%)	1 (2.56%)	
4	0 (0.00%)	1 (2.56%)	1 (2.56%)	
Treatments				
Antihypertensives	7 (17.9%)	10 (25.6%)	11 (28.2%)	0.380
cDMARD	8 (20.5%)	7 (17.9%)	3 (7.69%)	0.104
bDMARD	1 (2.56%)	2 (5.13%)	3 (7.69%)	0.548
Antimalarials	33 (84.6%)	26 (66.7%)	29 (74.4%)	0.459
Tacrolimus	0 (0.00%)	0 (0.00%)	1 (2.56%)	0.849
Cyclosporine: 0	39 (100%)	39 (100%)	39 (100%)	
Azathioprine	3 (7.69%)	8 (20.5%)	7 (17.9%)	0.615
Cyclophosphamide: 0	39 (100%)	39 (100%)	39 (100%)	
Treatment				0.257
Non-IS	26 (66.7%)	20 (51.3%)	17 (43.6%)	
IS	13 (33.3%)	19 (48.7%)	22 (56.4%)	
AGEs				
Skin AGEs	2.41 (0.59)	2.57 (0.57)	2.65 (0.76)	0.242
Skin AGEs assessment				0.233
<1SD	3 (7.69%)	2 (5.13%)	0 (0.00%)	
1SD-Mean	6 (15.4%)	4 (10.3%)	3 (7.69%)	
Mean	1 (2.56%)	2 (5.13%)	1 (2.56%)	
Mean->1SD	13 (33.3%)	9 (23.1%)	12 (30.8%)	
>1SD	16 (41.0%)	22 (56.4%)	23 (59.0%)	
Pentosidine	1304 [970;1721]	1372 [1047;1797]	1408 [1182;1871]	0.372
sRAGE	359 [245;514]	716 [534;847]	476 [354;676]	0.859

Supplementary Table S5: Non-significant (p-value >0.1) demographic and disease characteristics of systemic lupus erythematosus patients and their distribution according to CML tertiles in the exploratory analysis. “c” indicates variables which have been categorized as previously stated in the methodology section. * Indicates values according to the blood test performed in the study. *ANA: antinuclear antibodies; APL: antiphospholipid; APS; antiphospholipid syndrome; SADs: systemic autoimmune diseases; CRP: C-reactive protein; ESR: erythrocyte sedimentation rate; RV: reference value; CH50, C3 and C4: Complement CH50, C3 and C4; IL-6: interleukin 6; DAS28: Disease activity score 28; SLEDAI: systemic lupus erythematosus disease activity index; SLE-DAS: systemic lupus erythematosus disease activity score; SDI: systemic lupus erythematosus damage index; PGA: physician global assessment; FACIT: functional assessment of chronic illness therapy; HAQ: health assessment questionnaire disability index; PtGA: patient global assessment; VAS: Visual analogic scale; CVRF: cardiovascular risk factors (obesity = IMC> 30 Kg/m2, arterial hypertension, dyslipidemia, chronic renal disease*

or hyperuricaemia); CVE: cardiovascular events (angina, myocardial infarction, cerebrovascular accident, peripheral arterial disease, intestinal ischemia or ischemia of some other territory); CVE_SDI: cardiovascular events assessed in the SLE damage index (cerebral vascular accident, pulmonary infarction, angina or coronary bypass, myocardial infarction, venous thrombosis or infarction of the gastrointestinal tract); cDMARD; Disease-modifying antirheumatic drugs; bDMARD: biological DMARD; IS: immunosuppressants; AGEs: advanced glycation end products; SD: standard deviation; sRAGE: receptor for advanced glycation end-products;

	OLS linear regression	Gamma GLM
**(Intercept)**	332.806 ***	332.806 ***
	(CI=[274.336, 391.276],p = 0.000)	(CI=[283.766, 381.845],p = 0.000)
**Non-Caucasian ethnicities**	144.618 **	144.618 *
	(CI=[42.021, 247.215],**p = 0.006**)	(CI=[31.953, 257.283],**p = 0.013**)
**(Intercept)**	244.217 ***	234.488 ***
	(CI=[174.929, 313.505],p = 0.000)	(CI=[181.650, 287.326],p = 0.000)
**Disease duration**	4.102 *	4.347 *
	(CI=[0.290, 7.914],**p = 0.035**)	(CI=[0.558, 8.135],**p = 0.026**)
**Non-Caucasian ethnicities**	100.640 *	128.964 **
	(CI=[14.939, 186.340],p = 0.022)	(CI=[39.714, 218.213],p = 0.005)
**Glucocorticoids**	153.386 **	202.328 **
	(CI=[62.856, 243.915],p = 0.001)	(CI=[84.720, 319.937],p = 0.001)
**(Intercept)**	281.586 ***	307.334 ***
	(CI=[217.110, 346.062],p = 0.000)	(CI=[244.084, 370.584],p = 0.000)
**Anti-dsDNA 2nd tertile [2, 11)**	92.224 *	66.477
	(CI=[1.042, 183.407],**p = 0.047**)	(CI=[-33.876, 166.829],p = 0.197)
**Anti-dsDNA 3r tertile [11,300]**	124.908 **	129.480 *
	(CI=[31.157, 218.660],**p = 0.009**)	(CI=[15.550, 243.410],**p = 0.028**)
**(Intercept)**	298.664 ***	300.312 ***
	(CI=[234.713, 362.615],p = 0.000)	(CI=[240.613, 360.011],p = 0.000)
**IL-6 2nd tertile [1.88, 3.24)**	-31.427	-14.590
	(CI=[-119.805, 56.951],p = 0.482)	(CI=[-97.111, 67.930],p = 0.730)
**IL-6 3d tertile [3.24,39.38]**	105.876 *	83.828
	(CI=[16.522, 195.231],**p = 0.021**)	(CI=[-16.035, 183.690],p = 0.103)
**Glucocorticoids**	129.405 **	231.524 ***
	(CI=[43.234, 215.577],p = 0.004)	(CI=[106.488, 356.559],p = 0.000)
**(Intercept)**	302.059 ***	299.238 ***
	(CI=[255.371, 348.747],p = 0.000)	(CI=[257.970, 340.505],p = 0.000)
**Densitometric osteoporosis**	103.270 *	136.359 *
	(CI=[0.093, 206.447],**p = 0.050**)	(CI=[11.680, 261.037],**p = 0.034**)
**Non-Caucasian ethnicities**	88.636 *	102.513 *
	(CI=[10.842, 166.431],p = 0.026)	(CI=[17.808, 187.218],p = 0.019)

Supplementary Table S6: Ordinary least squares linear regression and gamma generalized linear model showing associations found between CML and systemic lupus erythematosus characteristics adjusted by their confounders (in grey). We only show the results that were statistically significant. Bold indicates those p-values significant (p<0.05). *** p < 0.001; ** p < 0.01; * p < 0.05. *Anti-dsDNA: anti-dsDNA antibodies; IL-6: interleukin 6.*

Variables	First tertile [0.823, 2.79)	Second tertile [2.793, 4.56)	Third tertile [4.564,31.68]	p-value
	N=38	N=37	N=16	
Gender: Female	35 (92.1%)	35 (94.6%)	16 (100%)	0.636
Age	53.2 (14.1)	52.4 (15.0)	51.6 (11.8)	0.702
BMI	26.3 (5.74)	24.9 (3.77)	25.3 (3.51)	0.413
Ethnicity				0.210
Caucasian	23 (60.5%)	26 (70.3%)	13 (81.2%)	
Other	15 (39.5%)	11 (29.7%)	3 (18.8%)	
Years of duration tertiles				0.144
[0, 5)	11 (28.9%)	8 (21.6%)	1 (6.25%)	
[5,16)	15 (39.5%)	17 (45.9%)	7 (43.8%)	
[16.45]	12 (31.6%)	12 (32.4%)	8 (50.0%)	
Classificatory Criteria and Other Clinical and Serological Data				
Constitutional symptoms	3 (7.89%)	4 (10.8%)	1 (6.25%)	0.046
Cutaneous	29 (76.3%)	28 (75.7%)	14 (87.5%)	0.714
Oral ulcer	18 (47.4%)	12 (32.4%)	10 (62.5%)	0.604
Alopecia	17 (44.7%)	18 (48.6%)	6 (37.5%)	0.513
Arthritis	30 (78.9%)	29 (78.4%)	14 (87.5%)	0.415
Serositis	2 (5.26%)	4 (10.8%)	2 (12.5%)	0.954
Renal	4 (10.5%)	2 (5.41%)	2 (12.5%)	0.881
Neurological	4 (10.5%)	4 (10.8%)	1 (6.25%)	0.576
Hematological	30 (78.9%)	28 (75.7%)	13 (81.2%)	0.625
ANA+ ever	38 (100%)	37 (100%)	16 (100%)	
Anti-dsDNA+ ever	23 (60.5%)	26 (70.3%)	14 (87.5%)	0.025
Anti-Sm+ ever	5 (13.2%)	6 (16.2%)	6 (37.5%)	0.142
Low complement	19 (50.0%)	19 (51.4%)	9 (56.2%)	0.475
Direct Coombs+	1 (5.26%)	3 (13.6%)	3 (21.4%)	0.853
Pulmonary	2 (5.26%)	1 (2.70%)	0 (0.00%)	0.403
Cardiac	0 (0.00%)	2 (5.41%)	1 (6.25%)	0.667
Raynaud	13 (34.2%)	14 (38.9%)	6 (37.5%)	0.434
APL antibodies carrier	9 (23.7%)	7 (18.9%)	6 (37.5%)	0.862
APS	2 (5.26%)	2 (5.41%)	0 (0.00%)	0.845
Other SADs	25 (65.8%)	23 (62.2%)	8 (50.0%)	0.663
Serological Variables				
CH50*	59.8 [52.4;73.6]	62.8 [57.4;69.0]	61.4 [55.4;70.8]	0.765
C4*	20.5 (8.69)	20.5 (7.51)	17.9 (7.15)	0.204
SLE Activity and Damage Indexes				
DAS28	2.11 [1.27;3.05]	2.25 [1.68;2.73]	2.39 [1.68;2.89]	0.345
SLEDAI	4.00 [0.00;7.50]	5.00 [2.00;6.00]	5.00 [3.50;9.00]	0.291
SLE-DAS	1.78 [0.82;5.84]	2.16 [0.82;4.93]	6.05 [3.60;7.94]	0.253
SDI	0.00 [0.00;1.00]	0.00 [0.00;1.00]	1.00 [0.00;1.25]	0.876
PGA	1.50 [1.00;2.00]	2.00 [1.00;3.00]	2.00 [1.00;3.00]	0.591
Patient Reported Outcomes				
FACIT	14.5 [10.0;23.8]	20.0 [16.0;29.0]	16.5 [10.8;26.8]	0.963
HAQ	0.25 [0.00;0.72]	0.62 [0.12;1.00]	0.38 [0.22;0.47]	0.937
PtGA	2.00 [1.00;4.75]	2.50 [1.00;5.00]	3.25 [2.00;5.00]	0.984
Pain VAS	2.00 [0.00;6.00]	3.00 [0.00;6.50]	3.00 [0.00;5.25]	0.526
Comorbidities and Cardiovascular Disease				
Hypertension	10 (26.3%)	8 (21.6%)	3 (18.8%)	0.547
Dyslipidemia	3 (7.89%)	4 (10.8%)	3 (18.8%)	0.669
Cardiovascular disease	2 (5.26%)	2 (5.41%)	1 (6.25%)	0.837
Chronic renal disease	0 (0.00%)	1 (2.70%)	0 (0.00%)	0.762
Hyperuricemia	2 (5.26%)	0 (0.00%)	0 (0.00%)	0.213
Obesity	10 (26.3%)	4 (10.8%)	2 (12.5%)	0.387
CVRF >0	16 (42.1%)	15 (40.5%)	6 (37.5%)	0.448
CVRF				0.383

Variables	First tertile [0.823, 2.79)	Second tertile [2.793, 4.56)	Third tertile [4.564,31.68]	p-value
0	22 (57.9%)	22 (59.5%)	10 (62.5%)	
1	8 (21.1%)	13 (35.1%)	5 (31.2%)	
2	7 (18.4%)	2 (5.41%)	0 (0.00%)	
3	1 (2.63%)	0 (0.00%)	1 (6.25%)	
CVE				0.673
0	33 (86.8%)	33 (89.2%)	15 (93.8%)	
1	4 (10.5%)	2 (5.41%)	0 (0.00%)	
2	1 (2.63%)	1 (2.70%)	0 (0.00%)	
3	0 (0.00%)	1 (2.70%)	1 (6.25%)	
CVE_SDI_presence	3 (7.89%)	4 (10.8%)	1 (6.25%)	0.823
CVRF&CVE >0	17 (44.7%)	15 (40.5%)	6 (37.5%)	0.407
CVRF&CVE				0.731
0	21 (55.3%)	22 (59.5%)	10 (62.5%)	
1	12 (31.6%)	11 (29.7%)	5 (31.2%)	
2	5 (13.2%)	2 (5.41%)	0 (0.00%)	
3	0 (0.00%)	1 (2.70%)	0 (0.00%)	
4	0 (0.00%)	1 (2.70%)	1 (6.25%)	
Treatments				
Dyslipidemia drugs	3 (7.89%)	6 (16.2%)	4 (25.0%)	0.332
Antihypertensives	10 (26.3%)	10 (27.0%)	3 (18.8%)	0.510
cDMARD	3 (7.89%)	8 (21.6%)	3 (18.8%)	0.550
bDMARD	0 (0.00%)	3 (8.11%)	3 (18.8%)	0.180
Antimalarials	29 (76.3%)	25 (67.6%)	14 (87.5%)	0.256
Tacrolimus	1 (2.63%)	0 (0.00%)	0 (0.00%)	0.179
Cyclosporine: 0	38 (100%)	37 (100%)	16 (100%)	
Azathioprine	6 (15.8%)	7 (18.9%)	4 (25.0%)	0.954
Cyclophosphamide: 0	38 (100%)	37 (100%)	16 (100%)	
AGEs				
Skin AGEs	2.46 (0.77)	2.60 (0.53)	2.74 (0.65)	0.877
Skin AGEs assessment				0.388
<1SD	5 (13.2%)	1 (2.70%)	1 (6.25%)	
1SD-Mean	5 (13.2%)	3 (8.11%)	1 (6.25%)	
Mean	2 (5.26%)	1 (2.70%)	0 (0.00%)	
Mean->1SD	13 (34.2%)	11 (29.7%)	3 (18.8%)	
>1SD	13 (34.2%)	21 (56.8%)	11 (68.8%)	
Pentosidine	1337 [1029;1740]	1363 [1146;1580]	1343 [1042;2536]	0.485
sRAGE	448 [335;671]	562 [433;852]	660 [546;977]	0.919

Supplementary Table S7: Non-significant (p-value >0.1) demographic and disease characteristics of systemic lupus erythematosus patients and their distribution according to CEL tertiles in the exploratory analysis. "c" indicates variables which have been categorized as previously stated in the methodology section. * Indicates values according to the blood test performed in the study. *BMI: body mass index; ANA: antinuclear antibodies; APL: antiphospholipid; APS; antiphospholipid syndrome; SADs: systemic autoimmune diseases; CH50 and C4: complement CH50 and C4; DAS28: disease activity score 28; SLEDAI: systemic lupus erythematosus disease activity index; SLE-DAS: systemic lupus erythematosus disease activity score; SDI: systemic lupus erythematosus damage index; PGA: physician global assessment; FACIT: functional assessment of chronic illness therapy; HAQ: health assessment questionnaire disability index; PtGA: patient global assessment; VAS: visual analogic scale; CVRF: cardiovascular risk factors (obesity = IMC> 30 Kg/m2, arterial hypertension, dyslipidemia, chronic renal disease or hyperuricaemia); CVE: cardiovascular events (angina, myocardial infarction, cerebrovascular accident, peripheral arterial disease, intestinal ischemia or ischemia of some other territory); CVE_SDI: cardiovascular events assessed in the SLE damage index (cerebral vascular accident, pulmonary infarction, angina or coronary bypass, myocardial infarction, venous thrombosis or infarction of the gastrointestinal tract);*

cDMARD; disease-modifying antirheumatic drugs; bDMARD: biological DMARD; AGEs: advanced glycation end products; SD: standard deviation; sRAGE: receptor for advanced glycation end-products.

	OLS linear regression	Gamma GLM
**(Intercept)**	1.873 ***	1.891 ***
	(CI=[0.851, 2.894],p = 0.000)	(CI=[0.908, 2.874],p = 0.000)
**Number of manifestations ever**	0.162 *	0.161 *
	(CI=[0.022, 0.302],**p = 0.024**)	(CI=[0.018, 0.303],**p = 0.030**)
**Smoker**	0.940 **	0.905 *
	(CI=[0.313, 1.568],p = 0.004)	(CI=[0.145, 1.664],p = 0.022)
**(Intercept)**	2.847 ***	2.813 ***
	(CI=[2.517, 3.176],p = 0.000)	(CI=[2.500, 3.127],p = 0.000)
**Anti-dsDNA ab titer (IU/mL)**	0.015 *	0.018
	(CI=[0.001, 0.030],**p = 0.042**)	(CI=[-0.000, 0.036],p = 0.058)
**Smoker**	0.886 **	0.907 *
	(CI=[0.242, 1.531],p = 0.008)	(CI=[0.136, 1.678],p = 0.024)
**(Intercept)**	2.606 ***	2.606 ***
	(CI=[2.144, 3.069],p = 0.000)	(CI=[2.213, 2.999],p = 0.000)
**Positive anti-dsDNA ever**	0.619 *	0.620 *
	(CI=[0.060, 1.178],**p = 0.030**)	(CI=[0.099, 1.141],**p = 0.022**)
**Smoker**	0.905 **	0.906 *
	(CI=[0.271, 1.539],p = 0.006)	(CI=[0.134, 1.678],p = 0.024)
**(Intercept)**	2.666 ***	2.576 ***
	(CI=[2.269, 3.062],p = 0.000)	(CI=[2.189, 2.963],p = 0.000)
**IL-6 (pg/mL)**	0.093 *	0.122 *
	(CI=[0.016, 0.170],**p = 0.019**)	(CI=[0.023, 0.222],**p = 0.018**)
**Smoker**	1.084 ***	1.059 **
	(CI=[0.458, 1.711],p = 0.001)	(CI=[0.334, 1.783],p = 0.005)
**(Intercept)**	2.543 ***	2.523 ***
	(CI=[2.085, 3.001],p = 0.000)	(CI=[2.052, 2.995],p = 0.000)
**CML (pg/mL)**	0.002 ***	0.002 **
	(CI=[0.001, 0.003],**p = 0.000**)	(CI=[0.001, 0.003],**p = 0.003**)

Supplementary Table S8: Ordinary least squares linear regression and gamma generalized linear model showing associations found between CEL and systemic lupus erythematosus characteristics adjusted by their confounders (in grey). We only show the results that were statistically significant. Bold indicates those p-values significant ($p<0.05$). *** $p < 0.001$; ** $p < 0.01$; * $p < 0.05$. *ab: antibodies; IL-6: interleukin 6; CML: Nξ-(carboxymethyl)lysine.*

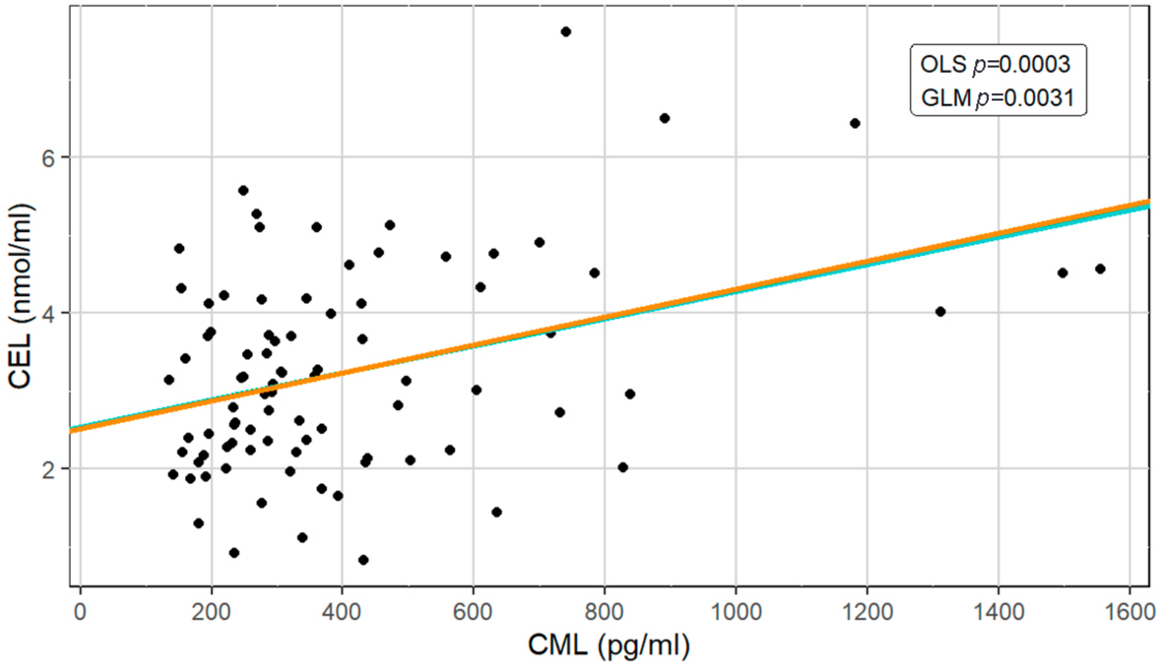

Supplementary Figure S2 Association between CEL and CML values. *CEL: Nξ-(carboxyethyl)lysine; CML: Nξ-(carboxymethyl)lysine; OLS: ordinary least squares; GLM: generalized linear model.*

Variables	**First tertile [122, 384)** *N=40*	**Second tertile [384, 671)** *N=40*	**Third tertile [671,2797]** *N=39*	**p-value**
Age	48.0 (14.5)	52.8 (16.1)	51.4 (13.8)	0.515
Body mass index	25.6 (5.67)	25.0 (3.67)	25.8 (4.87)	0.401
Ethnicity				0.494
Caucasian	24 (60.0%)	30 (75.0%)	27 (69.2%)	
Latin	10 (25.0%)	7 (17.5%)	10 (25.6%)	
Other	6 (15.0%)	3 (7.50%)	2 (5.13%)	
Years of duration tertiles				0.266
[0, 5)	20 (50.0%)	12 (30.0%)	10 (25.6%)	
[5,16)	14 (35.0%)	12 (30.0%)	15 (38.5%)	
[16.45]	6 (15.0%)	16 (40.0%)	14 (35.9%)	
Smoker	9 (22.5%)	11 (27.5%)	12 (30.8%)	0.782
Classificatory Criteria and Other Clinical and Serological Data				
Constitutional symptoms	3 (7.50%)	5 (12.5%)	3 (7.69%)	0.526
Cutaneous	31 (77.5%)	32 (80.0%)	26 (66.7%)	0.383
Oral ulcers	15 (37.5%)	19 (47.5%)	16 (41.0%)	0.624
Alopecia	18 (45.0%)	22 (55.0%)	14 (35.9%)	0.358
Arthritis	28 (70.0%)	30 (75.0%)	33 (84.6%)	0.205
Serositis	5 (12.5%)	1 (2.50%)	4 (10.3%)	0.509
Renal	3 (7.50%)	1 (2.50%)	4 (10.3%)	0.866
Neurological	4 (10.0%)	4 (10.0%)	2 (5.13%)	0.666
Hematological	29 (72.5%)	27 (67.5%)	29 (74.4%)	0.583
ANA+ ever	40 (100%)	40 (100%)	39 (100%)	
Anti-dsDNA+ ever	25 (62.5%)	21 (52.5%)	29 (74.4%)	0.171
Anti-Sm+ ever	8 (20.0%)	5 (12.5%)	8 (20.5%)	0.970
Anti-Ro60+ ever	18 (45.0%)	15 (37.5%)	15 (38.5%)	0.704
Anti-Ro52+ ever	11 (27.5%)	7 (17.5%)	9 (23.1%)	0.998
Low complement	21 (52.5%)	22 (55.0%)	20 (51.3%)	0.483
Direct Coombs+	2 (9.52%)	3 (10.7%)	4 (20.0%)	0.105
Pulmonary	2 (5.00%)	2 (5.00%)	1 (2.56%)	0.342
Cardiac	0 (0.00%)	2 (5.00%)	2 (5.13%)	0.164
Raynaud	14 (35.0%)	13 (33.3%)	12 (30.8%)	0.357
APL antibodies carrier	9 (22.5%)	13 (32.5%)	7 (17.9%)	0.628
Other SADs	22 (55.0%)	24 (60.0%)	29 (74.4%)	0.840
Serological Variables				
CRP*	0.14 [0.08;0.46]	0.16 [0.10;0.30]	0.12 [0.06;0.27]	0.209
AntidsDNA>RV*	11 (27.5%)	10 (25.0%)	15 (39.5%)	0.393
CH50*	58.2 [44.0;71.3]	60.3 [54.6;71.0]	62.2 [53.5;70.4]	0.167
C3*	106 (23.0)	104 (18.9)	108 (26.0)	0.563
C4*	19.4 (9.43)	19.5 (7.32)	20.6 (8.20)	0.904
IL-6>RV*	7 (18.4%)	4 (10.0%)	3 (7.89%)	0.198
SLE Activity and Damage Indexes				
SLEDAI	4.00 [1.50;6.00]	4.00 [2.00;6.00]	4.00 [2.00;7.00]	0.255
SLE-DAS	2.53 [1.20;6.85]	3.59 [1.57;5.10]	3.50 [1.57;5.66]	0.616
SDI	0.00 [0.00;1.00]	0.00 [0.00;1.00]	0.00 [0.00;1.00]	0.421
PGA	2.00 [1.00;2.00]	2.00 [1.00;3.00]	2.00 [1.00;3.00]	0.928
Patient Reported Outcomes				
FACIT	16.0 [10.0;27.0]	19.0 [11.5;28.0]	15.0 [10.5;25.5]	0.342
cHAQ				0.105
Normal (<0.3)	23 (57.5%)	18 (45.0%)	16 (42.1%)	
Mild (<1.3)	12 (30.0%)	17 (42.5%)	21 (55.3%)	
Moderate (<1.8)	4 (10.0%)	1 (2.50%)	1 (2.63%)	
Serious	1 (2.50%)	4 (10.0%)	0 (0.00%)	
PtGA	2.50 [1.00;4.25]	2.75 [1.00;5.00]	3.00 [1.00;5.00]	0.771
Comorbidities and Cardiovascular Disease				

Variables	First tertile [122, 384)	Second tertile [384, 671)	Third tertile [671,2797]	p-value
Hypertension	7 (17.5%)	9 (22.5%)	10 (25.6%)	0.777
Dyslipidemia	3 (7.50%)	5 (12.5%)	4 (10.3%)	0.466
Cardiovascular disease	0 (0.00%)	4 (10.0%)	1 (2.56%)	0.777
Chronic renal disease	1 (2.50%)	1 (2.50%)	1 (2.56%)	0.782
Hyperuricemia	2 (5.00%)	0 (0.00%)	0 (0.00%)	0.247
Obesity	9 (22.5%)	6 (15.0%)	7 (17.9%)	0.352
CVRF >0	16 (40.0%)	15 (37.5%)	16 (41.0%)	0.964
CVRF				0.947
0	24 (60.0%)	25 (62.5%)	23 (59.0%)	
1	11 (27.5%)	11 (27.5%)	11 (28.2%)	
2	4 (10.0%)	2 (5.00%)	4 (10.3%)	
3	1 (2.50%)	2 (5.00%)	1 (2.56%)	
CVE				0.407
0	35 (87.5%)	35 (87.5%)	38 (97.4%)	
1	4 (10.0%)	3 (7.50%)	0 (0.00%)	
2	1 (2.50%)	1 (2.50%)	0 (0.00%)	
3	0 (0.00%)	1 (2.50%)	1 (2.56%)	
CVRF&CVE >0	17 (42.5%)	15 (37.5%)	16 (41.0%)	0.854
CVRF&CVE				0.537
0	23 (57.5%)	25 (62.5%)	23 (59.0%)	
1	12 (30.0%)	10 (25.0%)	15 (38.5%)	
2	5 (12.5%)	3 (7.50%)	0 (0.00%)	
3	0 (0.00%)	1 (2.50%)	0 (0.00%)	
4	0 (0.00%)	1 (2.50%)	1 (2.56%)	
Treatments				
Dyslipidemia drugs	5 (12.5%)	6 (15.0%)	3 (7.69%)	0.163
Antihypertensives	7 (17.5%)	11 (27.5%)	10 (25.6%)	0.834
cDMARD	6 (15.0%)	7 (17.5%)	5 (12.8%)	0.386
Tacrolimus	0 (0.00%)	1 (2.50%)	0 (0.00%)	0.630
Cyclosporine: 0	40 (100%)	40 (100%)	39 (100%)	
Cyclophosphamide: 0	40 (100%)	40 (100%)	39 (100%)	
AGEs				
Skin AGEs	2.42 (0.67)	2.57 (0.61)	2.63 (0.67)	0.867
Skin AGEs assessment				0.574
<1SD	2 (5.00%)	3 (7.50%)	1 (2.56%)	
1SD-Mean	5 (12.5%)	3 (7.50%)	5 (12.8%)	
Mean	2 (5.00%)	0 (0.00%)	2 (5.13%)	
Mean->1SD	12 (30.0%)	13 (32.5%)	9 (23.1%)	
>1SD	19 (47.5%)	21 (52.5%)	22 (56.4%)	
CML	233 [175;435]	335 [225;462]	301 [277;402]	0.859
CEL	2.57 [2.17;3.99]	3.15 [2.07;4.12]	3.25 [2.62;4.51]	0.919
Pentosidine	1452 [1029;1737]	1368 [1040;1934]	1304 [1090;1710]	0.342

Supplementary Table S9: Non-significant (p-value >0.1) demographic and disease characteristics of systemic lupus erythematosus patients and their distribution according to sRAGE tertiles in the exploratory analysis. "c" indicates variables which have been categorized as previously stated in the methodology section. * Indicates values according to the blood test performed in the study. *ANA: antinuclear antibodies; APL: antiphospholipid; SADs: systemic autoimmune diseases; CRP: C-reactive protein; RV: reference value; CH50, C3 and C4: Complement CH50, C3 and C4; IL-6: interleukin 6; DAS28: Disease activity score 28; SLEDAI: systemic lupus erythematosus disease activity index; SLE-DAS: systemic lupus erythematosus disease activity score; SDI: systemic lupus erythematosus damage index; PGA: physician global assessment; FACIT: functional assessment of chronic illness therapy; HAQ: health assessment questionnaire disability index; PtGA: patient global assessment; VAS: visual analogic scale; CVRF: cardiovascular risk factors (obesity = IMC> 30 Kg/m2, arterial*

hypertension, dyslipidemia, chronic renal disease or hyperuricaemia); CVE: cardiovascular events (angina, myocardial infarction, cerebrovascular accident, peripheral arterial disease, intestinal ischemia or ischemia of some other territory); CVE_SDI: cardiovascular events assessed in the SLE Damage Index (cerebral vascular accident, pulmonary infarction, angina or coronary bypass, myocardial infarction, venous thrombosis or infarction of the gastrointestinal tract); cDMARD; disease-modifying antirheumatic drugs; CML: Nξ-(carboxymethyl)lysine; CEL: Nξ-(carboxyethyl)lysine; AGEs: advanced glycation end products; SD: standard deviation; sRAGE: receptor for advanced glycation end-products.

	OLS linear regression	Gamma GLM
**(Intercept)**	675.659 ***	661.469 ***
	(CI=[580.002, 771.315],p = 0.000)	(CI=[575.059, 747.880],p = 0.000)
**Gender Male**	-258.777 **	-278.148
	(CI=[-442.601, -74.952],**p = 0.007**)	(CI=[-570.268, 13.973],p = 0.062)
**Glucocorticoids**	-146.875	-149.658
	(CI=[-297.280, 3.529],p = 0.058)	(CI=[-318.314, 18.998],p = 0.081)
**(Intercept)**	549.351 ***	566.784 ***
	(CI=[420.658, 678.043],p = 0.000)	(CI=[458.581, 674.987],p = 0.000)
**Photosensitivity ever**	171.764 *	133.005 *
	(CI=[20.756, 322.772],p = 0.026)	(CI=[1.860, 264.150],p = 0.049)
**Gender Male**	-194.269	-223.367 **
	(CI=[-483.549, 95.012],**p = 0.186**)	(CI=[-385.526, -61.209],**p = 0.008**)
**Corticosteroid**	-188.862 *	-124.170
	(CI=[-354.068, -23.655],p = 0.025)	(CI=[-255.604, 7.263],p = 0.067)
**(Intercept)**	629.742 ***	624.089 ***
	(CI=[549.521, 709.964],p = 0.000)	(CI=[543.477, 704.702],p = 0.000)
**bDMARD**	700.624 ***	646.896 *
	(CI=[378.761, 1022.487],**p = 0.000**)	(CI=[93.627, 1200.166],**p = 0.024**)
**Glucocorticoids**	-239.162 **	-210.665 **
	(CI=[-401.550, -76.774],p = 0.004)	(CI=[-336.505, -84.826],p = 0.001)
**(Intercept)**	604.578 ***	599.723 ***
	(CI=[520.249, 688.908],p = 0.000)	(CI=[524.638, 674.807],p = 0.000)
**Mycophenolic acid**	361.566 ***	314.785 **
	(CI=[167.792, 555.340],**p = 0.000**)	(CI=[103.233, 526.337],**p = 0.004**)
**Glucocorticoids**	-241.104 **	-198.645 **
	(CI=[-408.067, -74.140],p = 0.005)	(CI=[-319.486, -77.805],p = 0.002)

Supplementary Table S10: Ordinary least squares linear regression and gamma generalized linear model showing associations found between sRAGE and systemic lupus erythematosus characteristics adjusted by their confounders (in grey). We only show the results that were statistically significant. Bold indicates those p-values significant (p<0.05). *** p < 0.001; ** p < 0.01; * p < 0.05. *bDMARD: biological disease-modifying antirheumatic drugs.*

Variables	First tertile [0, 1878)	Second tertile [1878, 3673)	Third tertile [3673,26506]	p-value
	N=39	*N=39*	*N=39*	
Years of duration	11.0 [5.00;15.0]	15.0 [3.00;21.0]	3.00 [1.00;12.0]	**0.096**
Anti-Ro52+ ever	8 (20.5%)	9 (23.1%)	10 (25.6%)	**0.062**
Pulmonary	0 (0.00%)	1 (2.56%)	4 (10.3%)	**0.048**
cTertile manifestations				**0.082**
[3, 7)	14 (35.9%)	15 (38.5%)	26 (66.7%)	
7	10 (25.6%)	9 (23.1%)	4 (10.3%)	
[8,12]	15 (38.5%)	15 (38.5%)	9 (23.1%)	
Anti-Ro52+*	6 (15.8%)	9 (23.1%)	10 (26.3%)	**0.028**
bDMARD	5 (12.8%)	1 (2.56%)	0 (0.00%)	**0.078**
AGEs	2.60 [2.15;2.90]	2.60 [2.05;3.05]	2.40 [1.95;2.60]	**0.099**
Pentosidine	1092 [833;1295]	1337 [1034;1640]	1875 [1480;2794]	**<0.001**
sRAGE	812 [660;1249]	456 [381;637]	315 [238;392]	**<0.001**

Supplementary Table S11: Variables that showed statistically significant (p-value <0.1) differences according to pentosidine/sRAGE tertiles in the exploratory analysis. "c" indicates variables which have been categorized as previously stated in the methodology section. * Indicates values according to the blood test performed in the study. *b-DMARD: biologic disease modifying antirheumatic-drugs; AGEs: advance glycation endo-products; sRAGE: receptor for advanced glycation end-products.*

	**OLS linear regression**	**Gamma GLM**
(Intercept)	3833.516 ***	3833.516 ***
	(CI=[3186.695, 4480.337],p = 0.000)	(CI=[3190.114, 4476.919],p = 0.000)
On bDMARD	-2561.386	-2561.386 ***
	(CI=[-5417.671, 294.899],p = 0.078)	(CI=[-3682.685, -1440.086],**p = 0.000)**
(Intercept)	3350.850 ***	3350.850 ***
	(CI=[2633.920, 4067.780],p = 0.000)	(CI=[2759.641, 3942.059],p = 0.000)
Anti-Ro52+*	1729.880 *	1729.880
	(CI=[192.236, 3267.524],**p = 0.028)**	(CI=[-70.782, 3530.542],p = 0.062)

Supplementary Table S12: Ordinary least squares linear regression and gamma generalized linear model showing associations found between pentosidine/sRAGE and systemic lupus erythematosus characteristics adjusted by their confounders (in grey). We only show the results that were statistically significant. Bold indicates those p-values significant ($p<0.05$). *** $p < 0.001$; ** $p < 0.01$; * $p < 0.05$. *Indicates values according to the blood test performed in the study. *bDMARD: biological disease-modifying antirheumatic drugs.*

Variables	First tertile [66.5, 432)	Second tertile [432.1, 827)	Third tertile [826.9,5194]	p-value
	N=39	*N=39*	*N=39*	
Ethnicity				**0.029**
Caucasian	30 (76.9%)	29 (74.4%)	20 (51.3%)	
Latin	7 (17.9%)	7 (17.9%)	13 (33.3%)	
Other	2 (5.13%)	3 (7.69%)	6 (15.4%)	
Ethnicity2				**0.010**
Caucasian	30 (76.9%)	29 (74.4%)	20 (51.3%)	
Other	9 (23.1%)	10 (25.6%)	19 (48.7%)	
GC	3 (7.69%)	8 (20.5%)	19 (48.7%)	**<0.001**
GC dosage	10.0 [8.75;11.2]	5.00 [4.38;10.0]	5.00 [2.50;8.75]	**0.091**
Anti-Ro60+ ever	16 (41.0%)	12 (30.8%)	20 (51.3%)	**0.018**
Anti-Ro52+ ever	7 (17.9%)	9 (23.1%)	11 (28.2%)	**0.026**
Anti-Ro52+*	5 (13.2%)	9 (23.7%)	11 (28.2%)	**0.011**
Hyperuricemia	0 (0.00%)	0 (0.00%)	2 (5.13%)	**0.041**
Densitometric OP	4 (10.3%)	4 (10.3%)	10 (25.6%)	**0.095**
Dyslipidemia drugs	2 (5.13%)	3 (7.69%)	9 (23.1%)	**0.021**
Anticoagulants	0 (0.00%)	0 (0.00%)	3 (7.69%)	**0.025**
CML	273 [185;295]	260 [205;345]	510 [379;736]	**<0.001**
sRAGE	778 [614;1220]	462 [358;626]	372 [265;466]	**<0.001**

Supplementary Table S123: Variables that showed statistically significant (p-value <0.1) differences according to CML/sRAGE tertiles in the exploratory analysis. "c" indicates variables which have been categorized as previously stated in the methodology section. * Indicates values according to the blood test performed in the study. *GC: glucocorticoids; OP: osteoporosis; CML: Nξ-(carboxymethyl)lysine; sRAGE: receptor for advanced glycation end-products.*

	**OLS linear regression**	**Gamma GLM**
**(Intercept)**	652.287 ***	652.287 ***
	(CI=[512.290, 792.283],p = 0.000)	(CI=[545.362, 759.212],p = 0.000)
**Ethnicity: non-Caucasian**	446.441 ***	446.441 **
	(CI=[201.842, 691.040],**p = 0.000**)	(CI=[167.125, 725.758],**p = 0.002**)
**(Intercept): SDI (sum=0)**	503.773 ***	510.213 ***
	(CI=[333.758, 673.787],p = 0.000)	(CI=[404.232, 616.195],p = 0.000)
**cSDI (sum=1)**	-30.764	29.278
	(CI=[-294.986, 233.458],p = 0.818)	(CI=[-163.422, 221.978],p = 0.766)
**cSDI (sum>1)**	358.614 *	314.662
	(CI=[35.570, 681.658],**p = 0.030**)	(CI=[-19.602, 648.926],p = 0.068)
**Glucocorticoids**	381.902 **	396.364 *
	(CI=[117.503, 646.301],p = 0.005)	(CI=[91.044, 701.684],p = 0.012)
**Ethnicity other**	451.710 ***	390.731 **
	(CI=[214.220, 689.201],p = 0.000)	(CI=[154.994, 626.467],p = 0.002)
**(Intercept)**	547.907 ***	564.080 ***
	(CI=[399.062, 696.751],p = 0.000)	(CI=[469.952, 658.209],p = 0.000)
**Densitometric osteoporosis**	508.853 **	456.089 **
	(CI=[198.100, 819.607],**p = 0.002**)	(CI=[117.263, 794.915],**p = 0.010**)
**EthnicityOther**	524.040 ***	492.828 ***
	(CI=[284.307, 763.772],p = 0.000)	(CI=[246.113, 739.544],p = 0.000)
**(Intercept)**	642.088 ***	653.954 ***
	(CI=[510.359, 773.817],p = 0.000)	(CI=[541.291, 766.617],p = 0.000)
**Dyslipidemia drugs**	387.215 *	307.681
	(CI=[29.425, 745.004],**p = 0.034**)	(CI=[-193.526, 808.888],p = 0.231)
**Glucocorticoids**	438.857 **	419.836 *
	(CI=[169.683, 708.031],p = 0.002)	(CI=[63.517, 776.155],p = 0.023)

Supplementary Table S14: Ordinary least squares linear regression and gamma generalized linear model showing associations found between CML/sRAGE and systemic lupus erythematosus characteristics adjusted by their confounders (in grey). We only show the results that were statistically significant. Bold indicates those p-values significant ($p<0.05$). *** $p < 0.001$; ** $p < 0.01$; * $p < 0.05$. *SDI: Systemic Lupus International Collaborating Clinics/American College of Rheumatology (SLICC/ACR) Damage Index; CML: Nξ-(carboxymethyl)lysine; sRAGE: receptor for advanced glycation end-products.*

Variables	First tertile [0.806, 3.77)	Second tertile [3.768, 7.20)	Third tertile [7.195,43.31]	p-value
	N=30	*N=30*	*N=30*	
GC	5 (16.7%)	5 (16.7%)	16 (53.3%)	0.036
Constitutional symptoms ever	1 (3.33%)	4 (13.3%)	3 (10.0%)	0.043
Anti-Ro52+ ever	5 (16.7%)	5 (16.7%)	11 (36.7%)	0.017
CRP	0.17 [0.07;0.32]	0.11 [0.07;0.21]	0.21 [0.12;0.47]	<0.001
CRP tertiles				0.015
[0.03,0.12)	11 (37.9%)	15 (50.0%)	6 (20.0%)	
[0.12,0.28)	10 (34.5%)	10 (33.3%)	10 (33.3%)	
[0.28,3.92]	8 (27.6%)	5 (16.7%)	14 (46.7%)	
ESR	10.0 [5.00;18.0]	8.00 [5.00;13.0]	13.0 [6.25;20.8]	0.045
Anti-dsDNA titers*	4.00 [1.00;11.0]	1.00 [1.00;12.8]	6.00 [2.00;34.5]	<0.001
AntiRo52+ *	4 (13.8%)	5 (17.2%)	10 (33.3%)	0.011
IL-6*	2.38 [1.75;4.06]	2.13 [1.48;2.91]	3.17 [1.90;4.43]	0.009
IL-6>RV*	1 (3.33%)	3 (10.0%)	6 (20.0%)	<0.001
Antimalarials	20 (66.7%)	21 (70.0%)	26 (86.7%)	0.036
NSAIDs	4 (13.3%)	2 (6.67%)	3 (10.0%)	0.042
CEL	2.30 [1.78;3.22]	2.90 [2.29;3.41]	4.12 [3.48;4.89]	<0.001
sRAGE	870 [614;1382]	561 [394;713]	378 [271;499]	<0.001

Supplementary Table S13: Variables that showed statistically significant (p-value <0.1) differences according to CEL/sRAGE tertiles in the exploratory analysis. "c" indicates variables which have been categorized as previously stated in the methodology section. * Indicates values according to the blood test performed in the study. *GC: glucocorticoids; CRP: C-reactive protein; ESR: erythrocyte sedimentation rate; IL-6: interleukin 6; RV: reference value; NSAIDs: non-steroid anti-inflammatory drugs; CEL: Nξ-(carboxyethyl)lysine; sRAGE: receptor for advanced glycation end-products.*

	**OLS linear regression**	**Gamma GLM**
**(Intercept)**	5.551 ***	5.514 ***
	(CI=[4.465, 6.638],p = 0.000)	(CI=[4.513, 6.516],p = 0.000)
**CRP**	2.607 *	2.800
	(CI=[0.113, 5.101],**p = 0.041**)	(CI=[-0.006, 5.606],p = 0.054)
**(Intercept)**	4.221 ***	4.485 ***
	(CI=[3.007, 5.436],p = 0.000)	(CI=[3.337, 5.633],p = 0.000)
**IL-6***	0.362 **	0.287
	(CI=[0.120, 0.604],**p = 0.004**)	(CI=[-0.040, 0.614],p = 0.089)
**Glucocorticoids**	3.274 ***	3.176 **
	(CI=[1.489, 5.059],p = 0.000)	(CI=[1.001, 5.351],p = 0.005)
**(Intercept)**	5.090 ***	4.842 ***
	(CI=[4.105, 6.075],p = 0.000)	(CI=[4.183, 5.501],p = 0.000)
**IL-6>RV***	3.440 *	3.544 *
	(CI=[0.739, 6.140],**p = 0.013**)	(CI=[0.212, 6.875],**p = 0.040**)
**Glucocorticoids**	3.121 ***	3.336 ***
	(CI=[1.331, 4.912],p = 0.001)	(CI=[1.485, 5.187],p = 0.001)

Supplementary Table S16: Ordinary least squares linear regression and gamma generalized linear model showing associations found between CEL/sRAGE and systemic lupus erythematosus characteristics adjusted by their confounders (in grey). We only show the results that were statistically significant. Bold indicates those p-values significant (p<0.05). *** p < 0.001; ** p < 0.01; * p < 0.05. *Indicates values according to the blood test performed in the study. *CRP: C-reactive protein; IL-6: interleukin 6; RV: reference value; CEL: Nξ-(carboxyethyl)lysine; sRAGE: receptor for advanced glycation end-products*

Variables	First tertile [0.858, 3.76)	Second tertile [3.761, 6.55)	Third tertile [6.550,17.74]	p-value
	N=40	*N=40*	*N=39*	
Gender: Female	39 (97.5%)	38 (95.0%)	34 (87.2%)	**0.027**
Age				0.114
<40	12 (30.0%)	11 (27.5%)	9 (23.1%)	
40-60	20 (50.0%)	21 (52.5%)	15 (38.5%)	
≥ 60	8 (20.0%)	8 (20.0%)	15 (38.5%)	
Years of duration	11.5 [4.00;16.0]	12.0 [4.75;20.0]	4.00 [1.00;13.5]	**0.088**
cYears duration				**0.059**
0-5	12 (30.0%)	14 (35.0%)	21 (53.8%)	
6-10	7 (17.5%)	5 (12.5%)	4 (10.3%)	
11-20	14 (35.0%)	12 (30.0%)	7 (17.9%)	
>20	7 (17.5%)	9 (22.5%)	7 (17.9%)	
Years of duration tertiles				**0.009**
[0, 5)	11 (27.5%)	10 (25.0%)	21 (53.8%)	
[5,16)	17 (42.5%)	14 (35.0%)	10 (25.6%)	
[16.45]	12 (30.0%)	16 (40.0%)	8 (20.5%)	
Photosensitivity ever	27 (67.5%)	26 (65.0%)	19 (48.7%)	**0.011**
GC	3 (7.50%)	13 (32.5%)	14 (35.9%)	**0.033**
SDI	0.00 [0.00;1.00]	0.00 [0.00;1.00]	0.00 [0.00;1.00]	**0.023**
APS	0 (0.00%)	1 (2.50%)	4 (10.3%)	**0.020**
APS or APL antibodies+	0.00 [0.00;0.00]	0.00 [0.00;1.00]	0.00 [0.00;1.00]	**0.069**
CRP	0.11 [0.07;0.26]	0.15 [0.12;0.26]	0.18 [0.08;0.44]	**0.078**
CRP tertiles				**0.086**
[0.03,0.12)	20 (51.3%)	9 (22.5%)	15 (38.5%)	
[0.12,0.28)	9 (23.1%)	21 (52.5%)	6 (15.4%)	
[0.28,3.92]	10 (25.6%)	10 (25.0%)	18 (46.2%)	
ANA+*	37 (94.9%)	37 (92.5%)	35 (89.7%)	**0.062**
IL-6>RV	2 (5.00%)	6 (15.4%)	6 (16.2%)	**0.049**
cHAQ				**0.006**
Normal (<0.3)	16 (40.0%)	19 (48.7%)	22 (56.4%)	
Mild (<1.3)	23 (57.5%)	16 (41.0%)	11 (28.2%)	
Moderate (<1.8)	1 (2.50%)	1 (2.56%)	4 (10.3%)	
Serious	0 (0.00%)	3 (7.69%)	2 (5.13%)	
CVE_SDI				**0.097**
0	40 (100%)	37 (92.5%)	33 (84.6%)	
1	0 (0.00%)	2 (5.00%)	4 (10.3%)	
2	0 (0.00%)	1 (2.50%)	2 (5.13%)	
CVE_SDI presence	0 (0.00%)	3 (7.50%)	6 (15.4%)	**0.059**
bDMARD	4 (10.0%)	2 (5.00%)	0 (0.00%)	**0.047**
Antimalarials	24 (60.0%)	32 (80.0%)	34 (87.2%)	**0.020**
Antiplatelet drugs	6 (15.0%)	15 (37.5%)	12 (30.8%)	**0.061**
Azathioprine	7 (17.5%)	9 (22.5%)	2 (5.13%)	**0.060**
AGEs	2.28 (0.50)	2.53 (0.68)	2.82 (0.66)	**<0.001**
Assessment				**0.045**
<1SD	3 (7.50%)	2 (5.00%)	1 (2.56%)	
1SD-Mean	7 (17.5%)	5 (12.5%)	1 (2.56%)	
Mean	2 (5.00%)	1 (2.50%)	1 (2.56%)	
Mean->1SD	12 (30.0%)	13 (32.5%)	9 (23.1%)	
>1SD	16 (40.0%)	19 (47.5%)	27 (69.2%)	
sRAGE	807 [701;1210]	521 [383;625]	301 [238;372]	**<0.001**

Supplementary Table S147: Variables that showed statistically significant (p-value <0.1) differences according to skin AGEs/sRAGE tertiles in the exploratory analysis. “c” indicates variables which have been categorized as previously stated in the methodology section. * Indicates values according to the blood test performed in the study. *GC: glucocorticoids; SDI: systemic lupus erythematosus damage index; APS: antiphospholipid syndrome; APL: antiphospholipid antibodies; CRP: C-reactive protein; ANA: antinuclear antibodies; RV: reference value; HAQ: health assessment questionnaire; CVE_SDI: cardiovascular events assessed in the SLE damage index (cerebral vascular accident, pulmonary infarction, angina or coronary bypass, myocardial infarction, venous thrombosis or infarction of the gastrointestinal tract); bDMARD: biologic disease-modifying antirheumatic drugs; AGEs: advanced glycation end-products; SD: standard deviation; sRAGE: receptor for advanced glycation end-products.*

	OLS linear regression	Gamma GLM
**(Intercept)**	5.286 ***	5.247 ***
	(CI=[4.498, 6.074],p = 0.000)	(CI=[4.527, 5.968],p = 0.000)
**Gender: Male**	2.854 *	3.069
	(CI=[0.176, 5.532],**p = 0.037**)	(CI=[-0.943, 7.082],p = 0.136)
**Glucocorticoids**	1.574 *	1.690
	(CI=[0.029, 3.118],p = 0.046)	(CI=[-0.105, 3.484],p = 0.068)
**(Intercept)**	6.6433 ***	6.8620 ***
	(CI=[5.621, 7.666],p = 0.0000)	(CI=[5.633, 8.0910],p = 0.0000)
**Years of duration 2nd tertile [5,16)**	-2.652 ***	-2.6390 ***
	(CI=[-4.075, -1.229],**p = 0.0003**)	(CI=[-4.099, -1.178],**p = 0.0006**)
**Years of duration 3rd tertile [16,45]**	-2.0700 **	-2.3861 **
	(CI=[-3.549, -0.591],**p = 0.0065**)	(CI=[-3.918, -0.854],**p = 0.0028)**
**Glucocorticoids**	2.0268 **	2.1690 **
	(CI=[0.647, 3.406],p = 0.0043)	(CI=[0.647, 3.691],p = 0.0061)

Supplementary Table S18: Ordinary least squares linear regression and gamma generalized linear model showing associations found between skin AGEs/sRAGE and systemic lupus erythematosus characteristics adjusted by their confounders (in grey). We only show the results that were statistically significant. Bold indicates those p-values significant (p<0.05). *** p < 0.001; ** p < 0.01; * p < 0.05. *RAGE: receptor for advanced glycation end-products.*
